# Dynamic response of the cell traction force to osmotic shock

**DOI:** 10.1038/s41378-023-00603-2

**Published:** 2023-10-16

**Authors:** Yongman Liu, Wenjie Wu, Shuo Feng, Ye Chen, Xiaoping Wu, Qingchuan Zhang, Shangquan Wu

**Affiliations:** 1 School of Biomedical Engineering, Anhui Medical University, 230032 Hefei, China; 2 CAS Key Laboratory of Mechanical Behavior and Design of Material, Department of Modern Mechanics, CAS Center for Excellence in Complex System Mechanics, University of Science and Technology of China, 230026 Hefei, China

**Keywords:** Nanobiotechnology, Optical physics

## Abstract

Osmotic pressure is vital to many physiological activities, such as cell proliferation, wound healing and disease treatment. However, how cells interact with the extracellular matrix (ECM) when subjected to osmotic shock remains unclear. Here, we visualize the mechanical interactions between cells and the ECM during osmotic shock by quantifying the dynamic evolution of the cell traction force. We show that both hypertonic and hypotonic shocks induce continuous and large changes in cell traction force. Moreover, the traction force varies with cell volume: the traction force increases as cells shrink and decreases as cells swell. However, the direction of the traction force is independent of cell volume changes and is always toward the center of the cell-substrate interface. Furthermore, we reveal a mechanical mechanism in which the change in cortical tension caused by osmotic shock leads to the variation in traction force, which suggests a simple method for measuring changes in cell cortical tension. These findings provide new insights into the mechanical force response of cells to the external environment and may provide a deeper understanding of how the ECM regulates cell structure and function.

**Figure:**
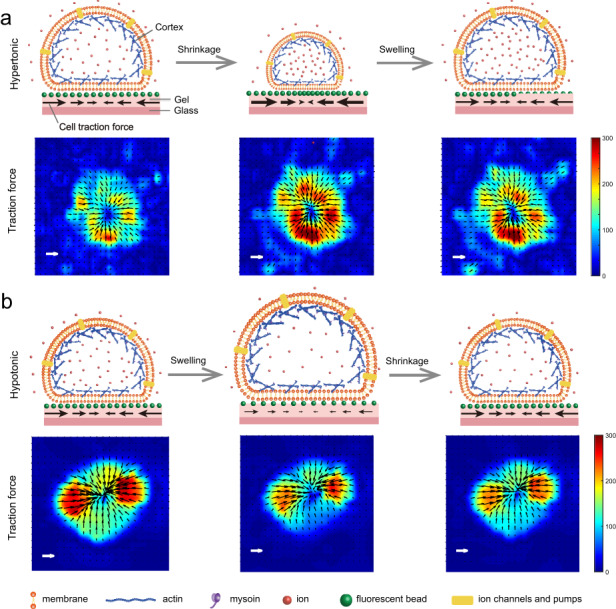
Traction force exerted by cells under hypertonic and hypotonic shocks. Scale bar, 200 Pa. Color bar, Pa. The black arrows represent the tangential traction forces.

Traction force exerted by cells under hypertonic and hypotonic shocks. Scale bar, 200 Pa. Color bar, Pa. The black arrows represent the tangential traction forces.

## Introduction

The extracellular environment is critical for many physiological processes, such as cell growth, cell proliferation and stem cell differentiation^1–3^. Cellular behaviors and functions are regulated by many external physical cues, such as osmotic pressure^4^, hydrostatic pressure^5^, matrix stiffness^6^, and mechanical force^7^. Among these, osmotic pressure has attracted the attention of many researchers. The structural and functional response of cells to osmotic pressure has been well studied, such as the effect of osmotic pressure on cell stiffness^4^, biofilm structure^8^, stem cell fate^4^, cell growth^9^, and cell viability^10^.

The mechanical force also plays an important role in many cellular processes in embryogenesis, such as cell proliferation, cell differentiation, and cell spatial rearrangements^4,7,11^. Stewart et al. found that the roundness of mitotic cells is derived from osmotic pressure and cortical tension^12^. Roffay et al. reported the relationship between cell volume and membrane tension during osmotic shock^13^. Some researchers have applied theoretical analyses to model the response of cortical tension to osmotic shock^14,15^. Cadart et al. reviewed the regulatory mechanisms of cell size across timescales, including the contribution of physical forces^16^. By imposing osmotic compressions on the extracellular matrix (ECM) embedded with cells, Dolega et al. studied the effects of selective and global compressions on cell volume, cell proliferation, and cell motility^17^. They found that the ECM acts as a pressure sensor and controls cell proliferation and migration^17^. The above studies are beneficial for understanding the mechanical response of cells to osmotic pressure, but studies on the measurement of force between cells and the ECM are still rare. Guo et al. studied the mechanical force generated by adherent mMSCs under osmotic compression, but they only measured the traction force at a certain time after the cell volume had stabilized, not the entire osmotic process^4^. The traction force exerted by cells under hypotonic shock is still lacking. Moreover, the dynamic evolution of the traction force during cell volume changes remains unclear. In addition, the fully adherent cells in their work were unable to recover after hypertonic shock. How cells interact with the ECM as they recover from osmotic shock is still unknown, and the underlying mechanical mechanism remains unclear. An in-depth understanding of how cells regulate the traction force during osmotic shock may provide new insights into cell mechanosensation and mechanotransduction. Therefore, quantitative characterization of the mechanical response of cells to external osmotic pressure is urgently needed.

Osmotic shocks cause water influx/efflux, leading to changes in cell volume and cortical tension^15^. Additionally, cells are tightly linked to the ECM by transmembrane proteins such as focal adhesions. Therefore, the cell volume changes induced by osmotic shock inevitably lead to the deformation of the ECM. In mechanics, deformation is often accompanied by a change in force. Namely, it is very likely that osmotic shock changes the mechanical force between cells and the ECM. The change in the cell-ECM force may induce tissue lesions and further lead to the onset and development of disease^18,19^. Therefore, an understanding of the development of the mechanical force between cells and the ECM during osmotic shock may provide insight into cell behavior and function as well as disease pathogenesis.

Here, we study the dynamic evolution of the mechanical interactions between cells and the ECM during osmotic shock by applying traction force microscopy to measure the traction force exerted by cells on the substrate. Furthermore, we propose a mechanical mechanism for the changes in traction force caused by osmotic shock and confirm it by drug perturbation experiments and numerical simulations.

## Results and discussion

The cell cortex, composed of actin filaments and associated proteins, is a thin network underlying the plasma membrane^20^. The shape of the slightly adherent cells is controlled by the cortical tension and the cell-substrate adhesion^21,22^. The former favors the formation of spherical cells, while the latter causes the spreading of individual cells. For simplicity, we treat the cell membrane and the cell cortex as a single structure^14^ and use the cortex and cortical tension to represent the structure and tension generated by the structure, respectively.

**Fig. 1:**
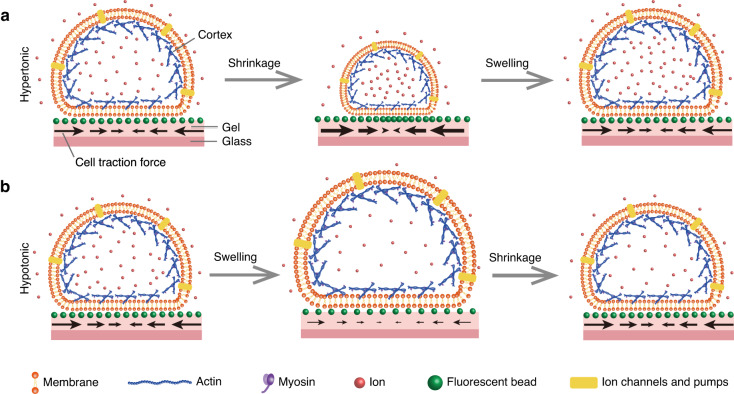
Schematic diagram of cells subjected to hypertonic and hypotonic solutions. The black arrows indicate the traction force exerted by cells on the substrate during osmotic shock

**Fig. 2:**
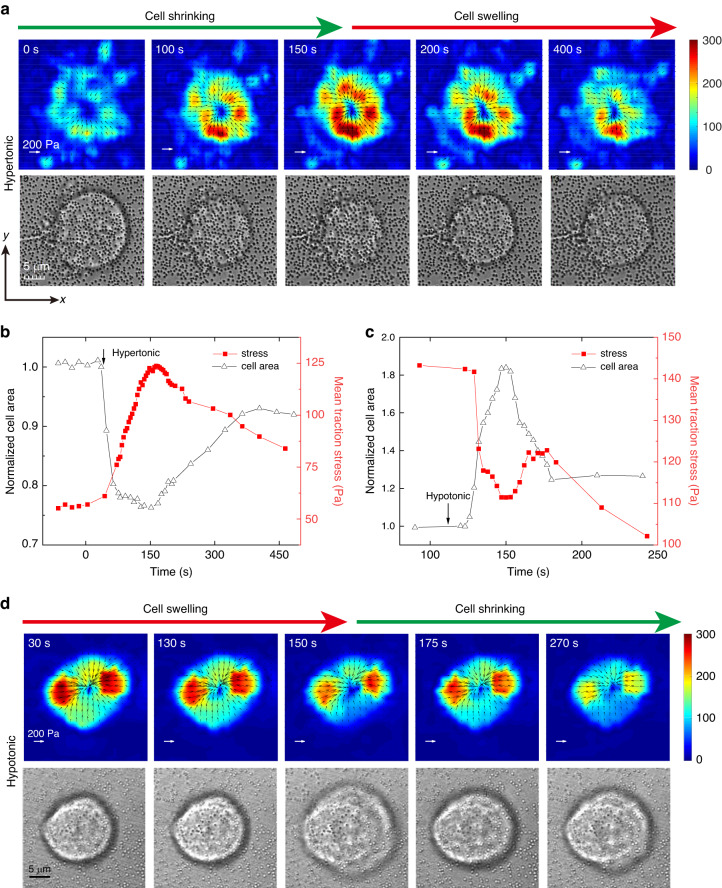
Time evolution of the traction force exerted by C2C12 cells subjected to osmotic shock. **a** Consecutive traction force maps exerted by a cell subjected to hypertonic solution. Normalized cell area and mean traction stress time courses for a cell subjected to hypertonic solution (**b**) and hypotonic solution (**c**). **d** Consecutive traction force maps applied by the cell in (**c**). Color bar, Pa. The black arrows in (**a**) and (**d**) represent the tangential traction forces (in-plane forces). The direction of the traction force was always inward for all cells during osmotic shock

We measured changes in traction force exerted by slightly adherent C2C12 cells on the substrate during osmotic shock. Slightly adherent cells (Figs. 1, S1, and S2) were obtained by controlling the culture time. Actin filaments formed and accumulated at the cell-substrate interface in the slightly adherent cells, applying and transmitting force to the substrate (see detailed discussion in section ‘Mechanical mechanism of the variation in traction force’). Before osmotic shock, inward traction stress was clearly observed on the substrate (Fig. 2a, d: 0–30 s), which indicated that the slightly adherent cells applied a detectable force. This result was consistent with a previous report that cells could apply a large force during the early stage of spreading^23^. The maximum traction force that cells applied in this study was approximately 280 Pa (Fig. 2d: 30 s), which was comparable to the force (500 Pa) exerted by fully adherent cells^24^. The observed traction force supported the presence of focal adhesions as they transmitted intracellular forces to the substrate.

**Fig. 3:**
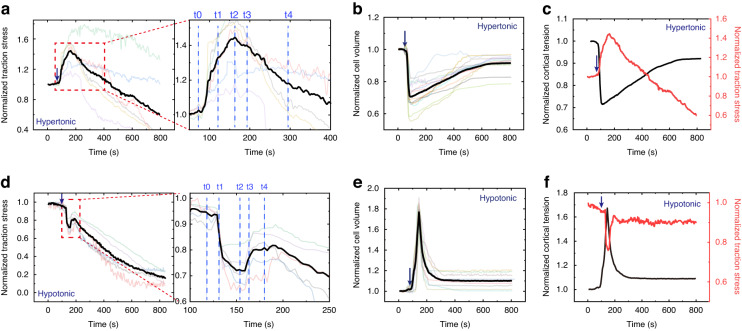
Mean traction stress and cortical tension time records for C2C12 cells subjected to osmotic shock. Normalized traction stress (**a**), normalized cell volume (**b**) and normalized cortical tension (**c**) for cells subjected to hypertonic shock. Normalized traction stress (**d**), normalized cell volume (**e**) and normalized cortical tension (**f**) time courses for cells subjected to hypotonic shock. To eliminate differences between the initial traction force of different cells, the traction force during osmotic shock was normalized. The mean traction stress and mean volume are shown in black

After hypertonic treatment (500 mOsm), the cells shrank immediately and continued to shrink for approximately 116 s (Fig. 2a, b: 36–160 s). During the shrinking process, water flowed out, and ions such as Na^+^ and K^+^ flowed into the cells, which reduced the hydrostatic pressure difference. Changes in cell volume usually led to changes in cortical tension. During osmotic shock, cell volume changed dramatically within tens of seconds (Fig. 2b), making it difficult to measure cortical tension directly and dynamically. To measure cortical tension dynamically, we used a spinning disk confocal microscope to measure cell volume during osmotic shock (Figs. 3b, e and S2) and then obtained the cortical tension (Fig. 3c, f) from cell volume according to a recent report (see supplementary information for membrane tension calculations)^13^. Our results showed that the cortical tension decreased as the cells shrank (Fig. 3b, c: 70–160 s), but the traction stress increased significantly (Fig. 3a: 70–160 s). In Fig. 2b, the mean traction stress increased from 58 Pa to 125 Pa. The decrease in cell volume caused by hypertonic shock activated ion transport systems (e.g., K^+^ and Cl^–^) in the cell membrane. These transport systems enabled the cells to uptake ions as well as water, resulting in an increase in volume toward the original value^25,26^. Therefore, cells began to recover after shrinkage and eventually reached an equilibrium state (Fig. 3b: 160–800 s). The final cell volume after recovery (Fig. 3b: reduced by approximately 10%) was slightly different from the initial value, in line with a previous report^9^. During recovery, traction stress continued to decrease (Fig. 3a: after 160 s), and cortical tension continued to increase (Fig. 3c: after 160 s). As shown in Fig. 2a, the traction stress peaked at 160 s (approximately 280 Pa) when the cell volume reached a minimum. The variation trend of traction stress during osmotic shock was opposite to that of cortical tension and cell volume: traction stress increased (decreased) as cell volume and cortical tension decreased (increased). After recovery, both cell volume and traction stress recovered almost to the initial values (Fig. 3a, b). Finally, the cell volume increased slowly, but the traction stress continued to decrease rapidly (Fig. 3a: 500–800 s). This behavior was probably due to the disruption of a few focal adhesions responsible for transmitting intracellular force to the substrate during cell volume changes.

To verify the response of the slightly adherent cells to hypertonic shock, we studied the effect of hyperosmolarity on another type of cell. After hypertonic shock, HepG2 cells shrank rapidly followed by slow recovery (Fig. S5 in the supplementary information), which was consistent with the above results. Furthermore, the traction stress generated by HepG2 cells during this process increased with cell shrinkage and decreased with cell swelling (Fig. S5 in the supplementary information). This suggested that the slightly adherent cells recovered from hypertonic shock and their traction force decreased (increased) as cell volume increased (decreased).

Under hypertonic shock, the traction stress first increased and then decreased, but its direction was always inward (Fig. 2a), suggesting that the direction of the traction stress was independent of cell volume changes. Additionally, both the magnitude and distribution of the traction corresponded to the deformation and shape of the cell (Fig. 2a). Figure 2a shows a significant increase in traction stress at the edge of the cell-substrate contacts, indicating the location of the maximum deformation. Furthermore, the magnitude of the traction stress was related to the distance from the center point. Along the radial direction (such as the *x*-axis) of the cell-substrate adhesion area, from the periphery to the center, the traction stress increased from zero to the maximum and then decreased to zero (Figs. 2a and 6c: hypertonic). The results also showed that traction stress varied rapidly with cell volume changes (Figs. 2a, b, 3a, b), suggesting that the mechanical response of cells to hypertonic shock was rapid.

To further investigate the mechanical response of cells to hypotonic shock, the time-lapse traction stress of a cell treated with hypotonic solutions (200 mOsm) is shown in Fig. 2c, d. Unlike hypertonic treatment, cells swelled immediately after hypotonic treatment due to the influx of water. Subsequently, cell volume recovered by shrinkage (Figs. 2c, d, 3e). Hypotonic-induced cell swelling activates specific ion channels that promote the loss of KCl and concomitant loss of water, leading to cell recovery^25,26^. The evolution of the traction stress under hypotonic shock was also different. After the addition of the hypotonic solution, cell volume and cortical tension increased rapidly, but traction stress decreased sharply (Fig. 3d–f: 100–160 s). During the recovery process, while cell volume decreased, traction stress gradually increased and cortical tension decreased (Fig. 3d–f: 160–200 s). The mean traction stress reached a minimum at 160 s when the cell volume and cortical tension were at a maximum. Finally, the cell volume was almost unchanged, and the final cell volume was approximately 110% of the initial area (Fig. 3e), consistent with a previous report^9^. After recovery, while the cell volume tended to be stable, the traction stress continued to decrease (Fig. 3d: after 200 s), possibly due to the partial detachment of the cell-matrix adhesion caused by the dramatic changes in cell volume. To eliminate signal interference caused by cell detachment, the baseline was removed from the traction force vs. time curve by polynomial fitting (Fig. 3f: red line). As shown in Fig. 3f, the traction stress decreased (increased) as the cortical tension increased (decreased). As with hypertonic stress, the direction of the traction stress was always inward (Fig. 2d). The distribution of the traction stress was also the same as that under hypertonic shock: from the periphery to the center, first increasing along the radial direction and then decreasing (Figs. 2d and 6c: hypotonic). Although the variation in traction force was different for hypotonic and hypertonic shocks, the relationships between traction force and cell volume were the same: traction force increased as cell volume decreased and decreased as cell volume increased (Fig. 3a, b, d, e). Furthermore, the traction force decreased (increased) with increasing (decreasing) cortical tension (Fig. 3c, f: before 200 s), suggesting that changes in traction force may correlate with changes in cortical tension.

The traction stress in this work was inverted from the displacement calculated by digital image correlation (DIC). According to the basic principle of DIC, the displacement was calculated from the deformed image (fluorescence image with cells) and the reference image (fluorescence image without cells) by a correlation algorithm. Our results (Figs. S3, S4) showed that regions with adherent cells deformed during osmotic shock, but no deformation was observed in regions without cells. These results indicated that osmotic shock did not cause deformation of the substrate, nor did it affect the traction force.

## Mechanical mechanism of the variation in traction force

A mechanical mechanism was proposed to explore how osmotic shock alters cell traction force. Cells were bound by the plasma membrane and the cortex. The cortex consisted of actin filaments and myosin, which generate contractile forces^27^. The cortical tension increased (decreased) with increasing (decreasing) cell volume (Fig. 3b, c, e, f). For cells adhering to the substrate, actin polymerized and accumulated during contact at the cell-substrate interface, formed a bundle and finally became a stress fiber during spreading^28^. The stress fiber was not visible until the cells have been cultured for 2–3 h^23^. The cell culture time in this paper was less than 1 h before imaging, which was much less than the time required for stress fibers to form. Thus, actin filaments, not stress fibers, accumulated at the cell-substrate interface. The actin filaments that accumulate at the cell-substrate interface are referred to as AF.

**Fig. 4:**
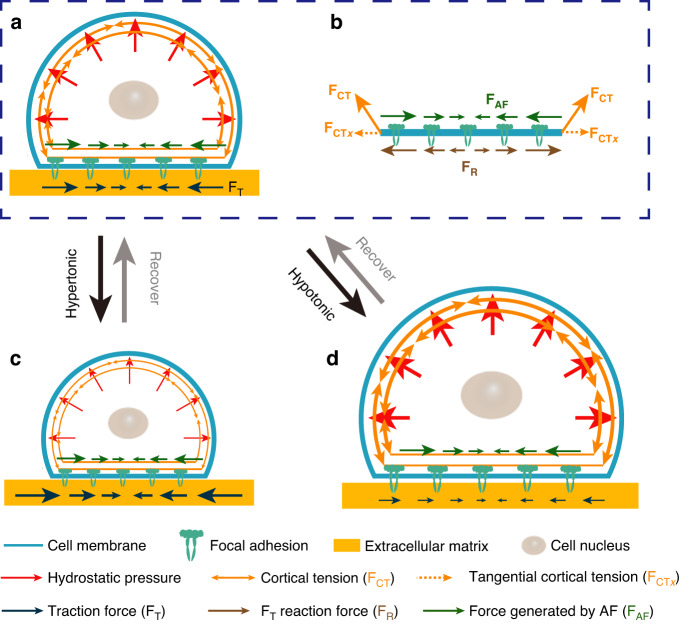
Cortical tension is responsible for traction force changes during osmotic shock. **a** Cells in an isotonic solution. **b** Cell cortex at the cell-substrate interface. **c** Cells under hypertonic shock. **d** Cells under hypotonic shock

Cells before osmotic shock were in an isotonic solution, as shown in Fig. 4a, b. The shape of the cells in Fig. 4 was based on the experimental results in Fig. S1. The cell cortex that contacted the substrate (Fig. 4b) reached mechanical equilibrium in the tangential direction (in plane) under the resultant action of three forces: the tangential component of the cortical tension (*F*_*CTx*_, yellow arrows), the contractile force applied by AF (*F*_*AF*_, green arrows), and the force from the substrate (*F*_*R*_, brown arrows). The tangential traction force on the substrate (black arrows, *F*_*T*_) was the reaction force of *F*_*R*_. Thus, *F*_*T*_ was determined by inward AF contraction (*F*_*AF*_, green arrows) and outward cortical tension (*F*_*CT*_, yellow arrows). Namely, *F*_*T*_ = *F*_*AF*_ – *F*_*CTx*_. Moreover, the traction force (*F*_*T*_, black arrows) on the substrate was large and always inward during osmotic shock (Fig. 2). Therefore, the inward force applied by the cells on the substrate (*F*_*AF*_, green arrows) was greater than the outward force (*F*_*CTx*_, yellow arrows).

Cells under hypertonic shock are shown in Fig. 4c. The hypertonic solution caused an efflux of water, leading to a decrease in both hydrostatic pressure differences across the cell cortex (red arrows in Fig. 4c) and cortical tension (*F*_*CT*_, yellow arrows in Fig. 4c). The traction force (*F*_*T*_, black arrows in Fig. 4c) on the substrate depended on the cell cortex and the AF accumulated at the cell-substrate contacts. While the cell cortex generates an outward force (*F*_*CTx*_, yellow arrows in Fig. 4c) on the substrate, the AF generates an inward force (*F*_*AF*_, green arrows in Fig. 4c) through contraction. After osmotic shock, cells deformed rapidly and returned to a steady state within a few minutes (Fig. 2). During this process, the traction force changed significantly in tens of seconds (Fig. 2). The force generated by the actin filaments (*F*_*AF*_) may not have changed in such a short time. Furthermore, when the traction force increased (decreased) significantly, the corresponding cortical tension showed a significant decrease (increase) (Fig. 3c: before 500 s). This suggested that cortical tension was the main cause of the variation in traction force. As shown in Fig. 4c, the reduction in cortical tension caused by the hypertonic solution led to an increase in traction stress (Fig. 3c: before 160 s). In the subsequent recovery process, cell volume increased, leading to an increase in cortical tension and further leading to a decrease in traction force (Fig. 3c: 160–500 s).

To further verify the proposed mechanism, the effect of hypotonic shock on traction force was analyzed (Fig. 4d). Hypotonic shock induced cell swelling, which increased cortical tension (*F*_*CT*_, yellow arrows in Fig. 4d, Fig. 3f: 100–160 s) and further led to a reduction in traction force (*F*_*T*_, black arrows in Fig. 4d, Fig. 3f: 100–160 s). After the initial swelling, the cells recovered from the hypotonic shock, leading to a decrease in cortical tension (*F*_*CT*_, Fig. 3f: 160–200 s) and an increase in traction force (*F*_*T*_). As the cell volume stabilized, the cortical tension gradually stabilized, and the corresponding traction force fluctuated within a small range (Fig. 3f: after 200 s). Moreover, when the variation in the cortical tension was large, the variation in the traction force at the same moment was also large (Fig. 3f: 100–200 s). This suggested that the variation in traction force (black arrows in Fig. 4d) during osmotic shock resulted from cortical tension changes (yellow arrows in Fig. 4d). Due to the rapidity and short duration of cellular deformation caused by osmotic shock, it was difficult to use traction force microscopy to capture the three-dimensional deformation exerted by cells on the substrate during this process. Recently developed astigmatic traction force microscopy (aTFM), which quantifies 3D cell traction force using single-frame astigmatic images rather than multiframe images, may be able to track three-dimensional deformations during osmotic shock^29^.

**Fig. 5:**
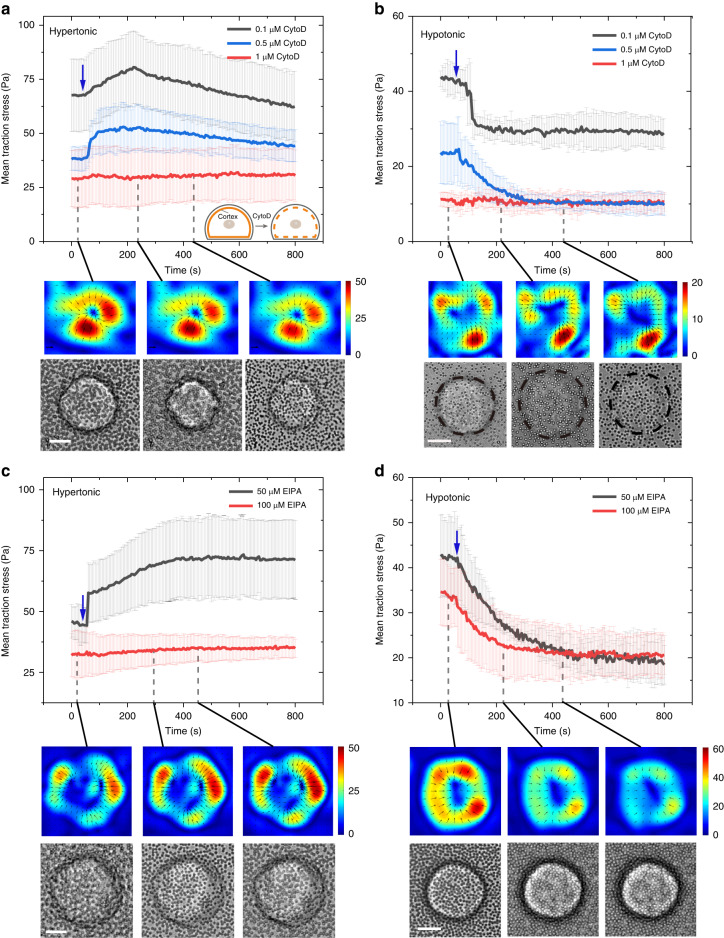
Mean traction stress time records for C2C12 cells subjected to molecular perturbations and osmotic shock. Mean traction stress exerted by cells treated with cytochalasin D (CytoD) under hypertonic shock (**a**) and hypotonic shock (**b**). The inset graphs in (**a**) show the effect of CytoD on the cell cortex (orange line). Mean traction stress exerted by cells treated with ethylisopropylamiloride (EIPA) under hypertonic shock (**c**) and hypotonic shock (**d**). Representative traction stress maps generated by cells exposed to high drug concentrations (red lines) are shown. The black arrows represent the tangential traction forces (in-plane forces). Scale bar, 5 μm. Color bar, Pa. The blue arrows indicate the onset of osmotic shock

To validate the proposed mechanism, experiments with inhibition of the cell cortex and ion transport were performed by adding cytochalasin D (CytoD) or ethylisopropylamiloride (EIPA) to cells before imaging. Figures 5a, b show the effect of CytoD, which inhibits actin polymerization, on cell traction force during osmotic shock. Consistent with untreated cells (Figs. 2a and d), the traction stress generated by the CytoD-treated cells was always directed toward the center of the cell-substrate interface during osmotic shock. At low concentrations (Figs. 5a, b: 0.1 μM), the traction stress first increased and then slowly recovered during hypertonic shock; it decreased but did not recover during hypotonic shock, possibly due to the disruption of actin filaments caused by CytoD. At high concentrations (Figs. 5a, b: 1 μM), the traction stress fluctuated within a small range for both hypertonic and hypotonic shocks, which was significantly different from the behavior of the untreated cells (Fig. 2). This behavior probably occurred because the cell cortex was disrupted by the high concentration of CytoD^30^, resulting in no large change in traction force during osmotic shock. In addition, the traction stress before osmotic shock decreased with increasing CytoD concentration. These results suggested that the changes in traction force during osmotic shock depended on actin and therefore on cortical tension, which was consistent with the mechanism proposed above.

The effect of EIPA, which inhibits Na^+^/H^+^ antiporters, was also tested on traction force during osmotic shock (Fig. 5c, d). Cells treated with EIPA underwent less swelling and shrinkage during osmotic shock than untreated cells (Figs. 2, 5c, d). The magnitude of the change in traction stress during this process was also significantly reduced (Figs. 2, 5c, d). This suggested that the change in traction stress during osmotic shock was caused by cell swelling or shrinkage.

Overall, osmotic shock inducesd changes in cell volume, which changed cortical tension and further altered traction force. Cell swelling caused by hypotonic shock induced an increase in cortical tension, reducing traction force. Cell shrinkage caused by hypertonic shock reduced cortical tension, which increased traction force. These results showed that the variation in traction force reflected changes in cortical tension.

## Numerical simulation

To validate the proposed mechanical mechanism, the traction force exerted by cells under osmotic shock was simulated using the finite element method (ABAQUS, version 2019, Dassault Systèmes, France). The cell was modeled as a spherical cap, and the substrate was modeled as a cylinder (Fig. S1c). The geometry of the cell in the model was based on experimental data (Fig. S1a, b). Before osmotic shock, the contact angle between the cell and the substrate was obtuse. Both the cell and the substrate were simplified as an isotropic linear elastic material. The tie surface constraints were applied on the cell-substrate interface. The bottom surface of the substrate was fixed. Other parameters used in the simulation are shown in Table S1 in the supplementary information.

Before osmotic shock, since ions and proteins were in the cytoplasm, the intracellular osmotic pressure was higher than that outside the cell. Therefore, there was a pressure difference across the cell cortex. In the simulation, the hydrostatic pressure difference was applied during osmotic shock on the cell cortex. The hydrostatic pressure caused by osmotic shock was obtained from a mathematical model of the cell cortex, which incorporated water permeation, ion transport and active stresses in the cortex^14^. Initially, the hydrostatic pressure difference was 100 Pa. Under hypertonic shock, the hydrostatic pressure difference was less than 100 Pa and toward the outer surface of the cell (80 Pa, 50 Pa, 60 Pa, and 90 Pa). Under hypotonic shock, the hydrostatic pressure difference was greater than 100 Pa and toward the outer surface of the cell (120 Pa, 150 Pa, 140 Pa, 130 Pa, 150 Pa).

**Fig. 6:**
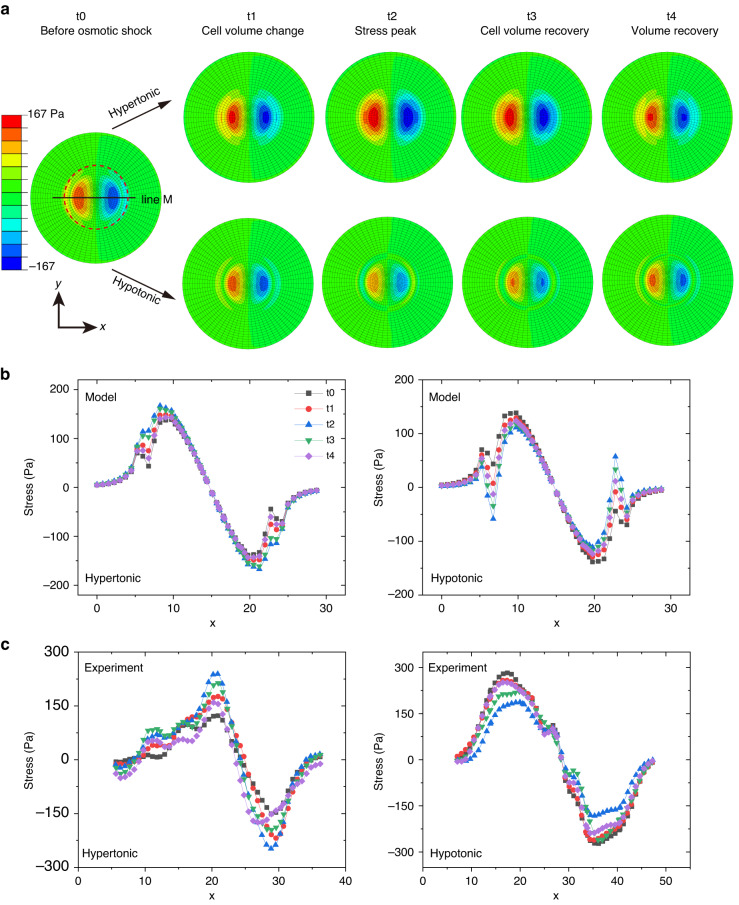
Comparison of the traction stress obtained from simulation and experiment. **a** Time series of the *x*-component of the stress (*σ*_*x*_) exerted on the substrate by cells subjected to osmotic shock. The red dotted line indicates the edge of the cell. **b** *σ*_*x*_ of points on line M in (**a**) obtained from finite element analysis. **c** *σ*_*x*_ of points on the line M in (**a**) obtained from the experiment

The simulation and experimental results were compared for some representative moments (cells at t0, t1, t2, t3, and t4 in Fig. 3) in Fig. 6. The traction stress along the *x* direction (*σ*_*x*_) obtained from the simulation is shown in Fig. 6a. Since the magnitude of *σ*_*x*_ theoretically related only to the distance to the center of the contact surface, line M passing through the center of the contact surface was used to analyze the time evolution of *σ*_*x*_, which reflected the traction stress on the whole substrate. The *σ*_*x*_ values of points on line M obtained from simulation (Fig. 6a) and experiment (Fig. 2) are shown in Fig. 6b, c, respectively. In Fig. 6b, there was a downward peak at *x* = 7 and an upward peak near *x* = 23. The reason for this behavior was that the tensile force generated by the cortex on the substrate (*F*_*CT*_ in Fig. 4) was concentrated at the edge of the cell-substrate contacts. This apparent stress concentration at the edge of the contacts occurred because a chamfer was not present on the cell-substrate contacts in the model. For the hypertonic treatment, the simulation results showed that *σ*_*x*_ increased as the cell shrank (from t0 to t2) and decreased as the cell swelled (from t2 to t4), which was consistent with the experimental results (Fig. 6c). Over time, the maximum variation in *σ*_*x*_ was approximately 45 Pa. For hypotonic treatment, the simulation results showed that *σ*_*x*_ decreased immediately as the cell swelled (from t0 to t2) and then increased as the cell shrank (t3), followed by a decrease after recovery (t4). In addition, the simulation results showed that as *x* increased, *σ*_*x*_ peaked in the positive direction and then decreased to zero, followed by an increase in the negative direction and finally a decrease (Fig. 6b), which was in line with the experimental results (Fig. 6c). The changing trend of *σ*_*x*_ during osmotic shock (from t0 to t4) obtained from the simulation was similar to that obtained from the experiment, suggesting that the proposed mechanical mechanism was reasonable.

Cell cortical tension is an important indicator for understanding cell function, and it is involved in many cellular processes, such as cell migration^31^, cell spreading^32^, phagocytosis^33^, and cell division^12^. Additionally, the cortical tension of cancer cells is higher than that of normal cells. Studying cortical tension is of great significance in physiology and clinical research because it provides insight into cancer. However, it is very difficult to measure cortical tension. Some researchers measure cortical tension by pulling membrane tubes out of the membrane^34,35^. Colom et al. developed a fluorescent lipid tension reporter to measure cell cortical tension by quantifying the fluorescence lifetime^36^. Popescu et al. used optical interferometry to quantify the thermal fluctuations of red blood cells and giant unilamellar vesicles and obtained their tension coefficient^37^. However, these methods are complex, expensive and usually rely on sophisticated technology (quantification of fluorescence changes or development of optical techniques). A simple, fast and inexpensive method for measuring the cortical tension of cells is urgently needed.

According to the results and findings presented above, the variation in cell traction force during osmotic shock resulted from the dynamic change in cell cortical tension. Our results showed that there was a quantitative relationship between cell traction force changes and cortical tension changes (Fig. 3c, f). By changing osmotic pressure and measuring cell traction force, it was possible to quantify changes in cell cortical tension. Thus, the results of this study provide a simple, low-cost and rapid method for measuring changes in cell cortical tension.

## Conclusions

In summary, the dynamic mechanical response of cells to osmotic shocks was quantified by measuring the cell traction force with high spatial and temporal resolution. The results showed that cell traction force decreased with increasing cell volume and increased with decreasing cell volume. Furthermore, this study confirmed that changes in the traction force during osmotic shock were mainly due to cortical tension changes. The results of this study not only indicated a simple and effective method to quantify changes in cell cortical tension but also contributed to a deeper understanding of how external mechanical stimuli affect cellular mechanotransduction.

### Supplementary information

Supplementary information: Dynamic response of cell traction force to osmotic shock

## Data Availability

All data supporting the results of this study are available in the paper and Supplementary Information. Other data are available from the corresponding author upon request.
