# Supplementary material for: Dynamic response of the cell traction force to osmotic shock
Source: Microsyst Nanoeng. 2023 Oct 16;9:131. doi: 10.1038/s41378-023-00603-2 (PMC10579240; doi:10.1038/s41378-023-00603-2)
Supplement: Supplementary file 1 — Supplementary information: Dynamic response of cell traction force to osmotic shock [file 41378_2023_603_MOESM1_ESM.pdf]

Supplementary information: Dynamic response of cell traction force to osmotic shock

Yongman Liu[1,2], Wenjie Wu[2], Shuo Feng[2], Ye Chen[2], Xiaopiing Wu[2], Qingchuan Zhang*[2], and Shangquan Wu*[2]

[1]School of Biomedical Engineering, Anhui Medical University, Hefei, 230032, China
[2]CAS Key Laboratory of Mechanical Behavior and Design of Material, Department of Modern Mechanics, CAS Center for Excellence in Complex System Mechanics, University of Science and Technology of China, Hefei, China

1 3D images of cells before and after osmotic shock

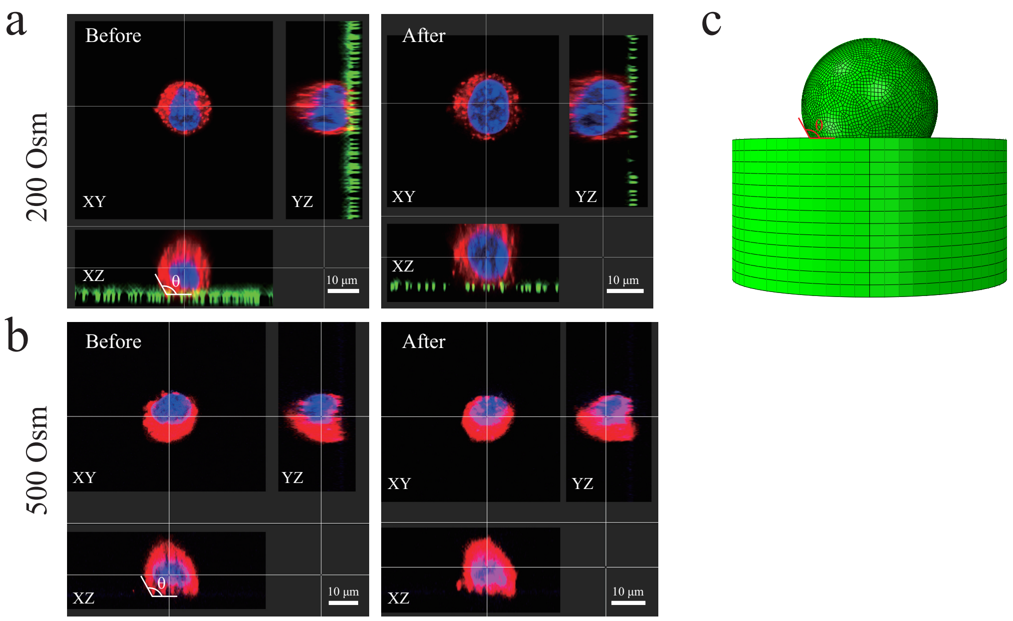

Figure S1: a, 3D image of a cell on the polyacrylamide gel with fluorescent beads (green) embedded before and after hypotonic shock. The total images dimensions were 100 × 100 × 30 μm. The cell membrane (red) and nuclei (blue) were stained with Dil and Hoechst 33258, respectively. A cell treated with the hypotonic solution. b, 3D image of a cell before and after hypertonic shock. c, Computational model of a cell on the substrate in finite element analysis.

Fig. S1a and S1b showed the 3D morphology of a cell before and after hypotonic and hypertonic shock, respectively. Cells were first captured before changing osmotic pressure (Fig. S1a,b: before) and then were captured again after the cell undergoing osmotic shock reached a steady state (Fig. S1a,b: after). The culture time of cells in Fig. S1 is the same as those in Fig. 3. The 3D images of cells on the substrate were obtained by laser scanning confocal microscope.

*Email: zhangqc@ustc.edu.cn; wushq@ustc.edu.cn

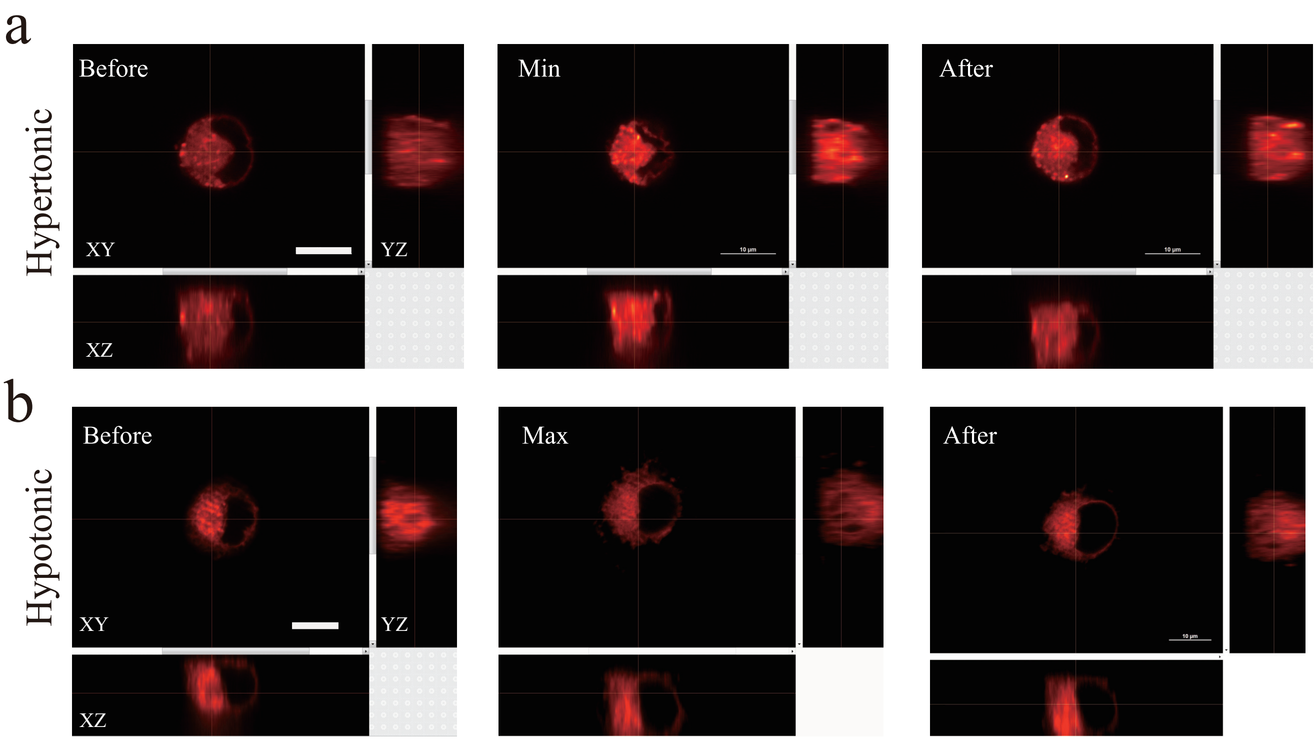

Figure S2: 3D images of cells captured by the spinning disk confocal microscope during hypertonic shock (a) and hypotonic shock (b). The cell membrane (red) were stained with Dil. Scale bar, 10 μm.

2 Successive displacement and traction stress contours exerted by a single cell subjected to osmotic shock

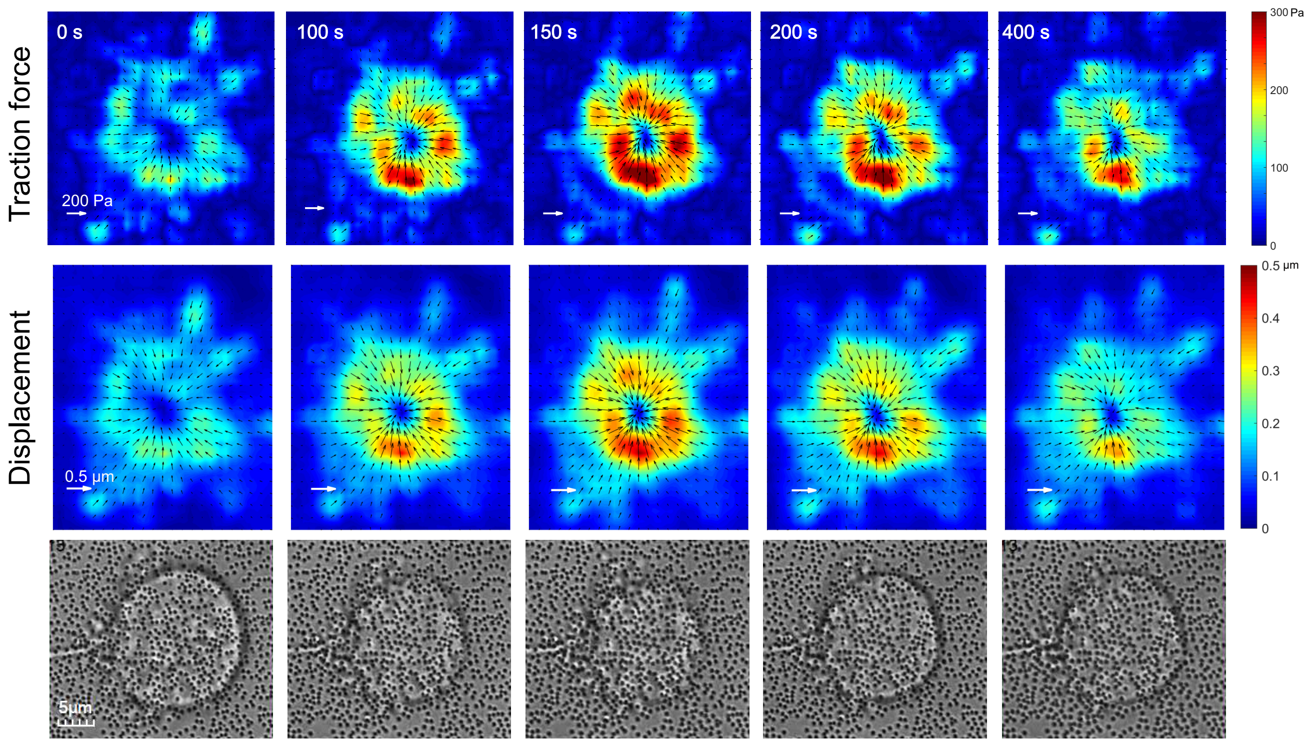

Figure S3: Time evolution of displacement and traction force contours of a single cell under hypertonic shock.

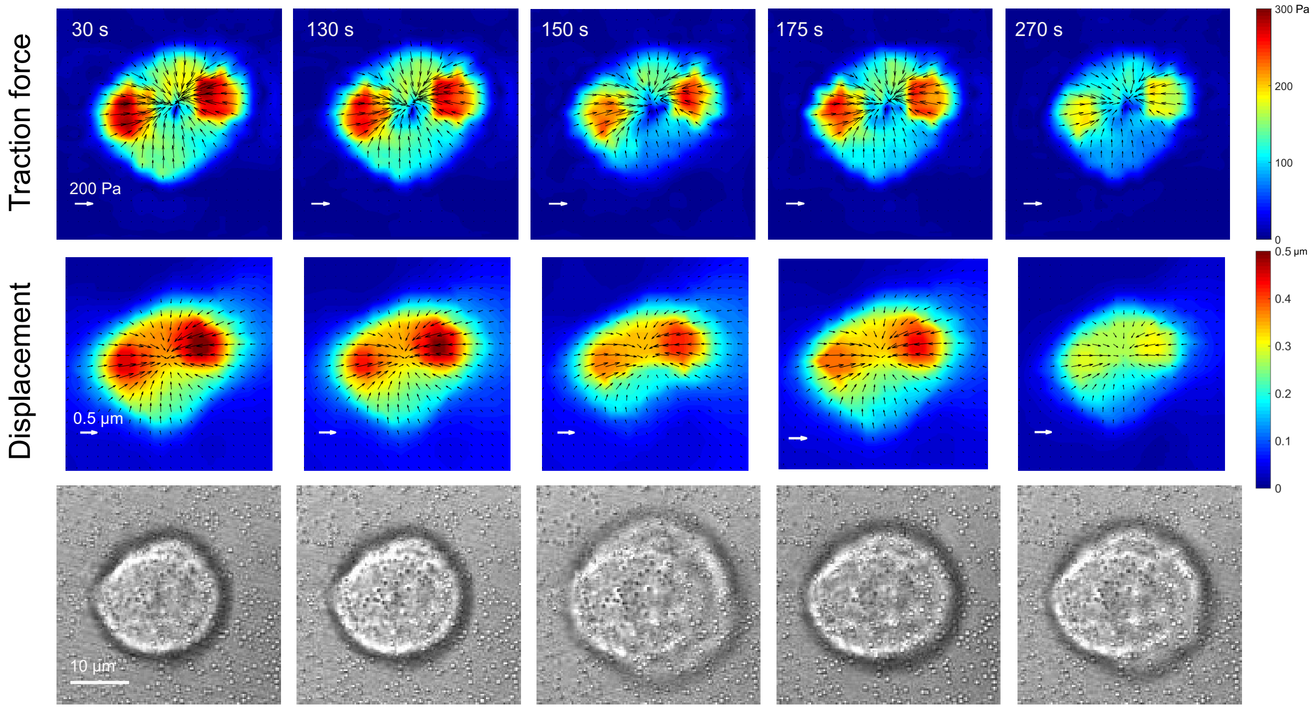

Figure S4: Time evolution of displacement and traction force contours of a single cell under hypotonic shock.

Cells were cultured on polyacrylamide (PAA) hydrogel embedded with fluorescent beads (Figure 1), as reported in previous work [1]. The Young's modulus of the PAA gel was 2.5 KPa [2]. Cells transmit force on the gel substrate through focal adhesion complexes, deforming the substrate. Time-lapse images were captured to record the movement of fluorescent beads embedded in the gel using laser scanning confocal microscopy. Based on the un-deformed image and the deformed image, the displacements of the substrate were calculated using digital image correlation. The traction force was reconstructed from the displacement and the constitutive relationship of the substrate according to the elastic mechanics theory [1, 3]. Fig. S3 and Fig. S4 showed the consecutive displacement and traction force contours on the substrate caused by a single C2C12 cell under hypertonic and hypotonic treatments, in respectively. Time-lapse images of the cell were captured every 3 seconds for 30 min.

Cells used in this paper were C2C12 cells unless otherwise stated.

Fig. S5 showed the relative changes in displacement and traction force on the substrate exerted by a HepG2 cell under hypertonic treatment. To calculate the relative displacements, the 3D images taken before hypertonic treatment (t=0 in Fig. S5) were used as the reference images in digital volume correlation. The relative traction stresses were obtained from the relative displacements. Cells shrank (Fig. S5b: 100-240 s) immediately after hypertonic shock. During shrinkage, cells generated an inward traction force compared to its initial state, and the inward force increased as cell volume decreased (Fig. S5a: 100-240 s). Cells then slowly recovered (Fig. S5: 240-540 s) by swelling until it was close to its initial volume. During recovery, the inward traction force gradually decreased and eventually increased in the opposite direction (outward in Fig. S5: 520 s). When the cell volume reached a minimum, the inward traction force reached a maximum (Fig. S5b). These results show that HepG2 cells are able to recover from hypertonic shock and their traction force increases with cell shrinkage and decreases with cell swelling, which is consistent with C2C12 cells (Fig. 2 and 3).

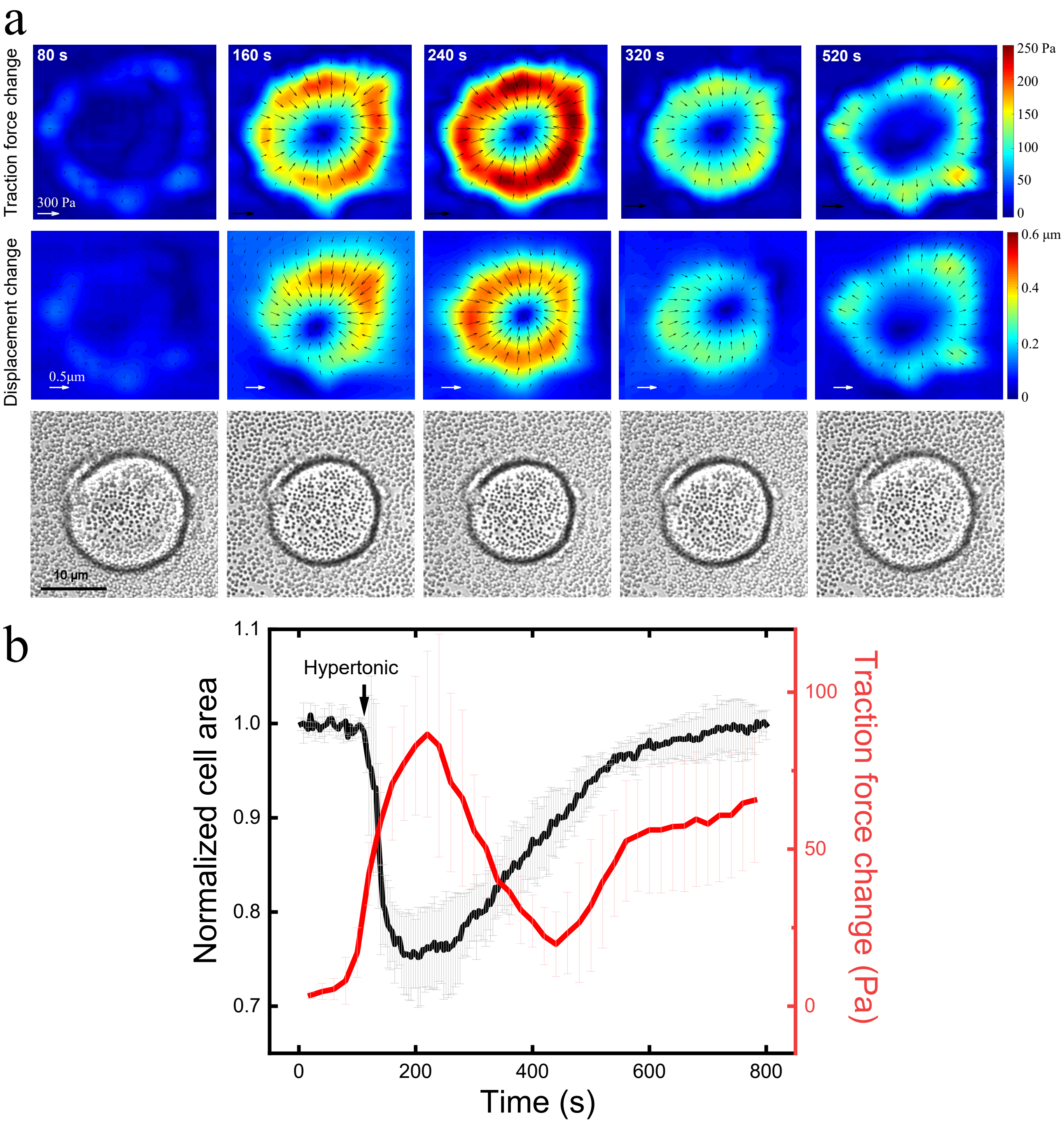

Figure S5: Relative change of displacement and traction stress of a HepG2 cell after hypertonic shock (a) and dynamics of the changes in cell area and mean traction force exerted by HepG2 cells (b), n=4.

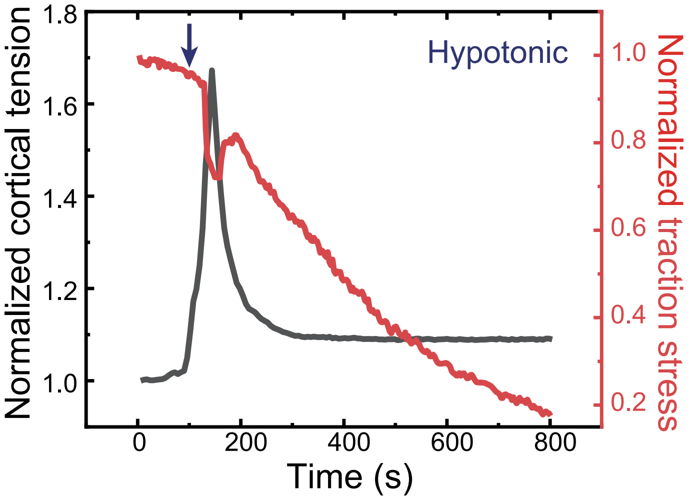

Figure S6: Time course of the cortical tension and the traction stress (original data) in cells exposed to hypotonic shock.

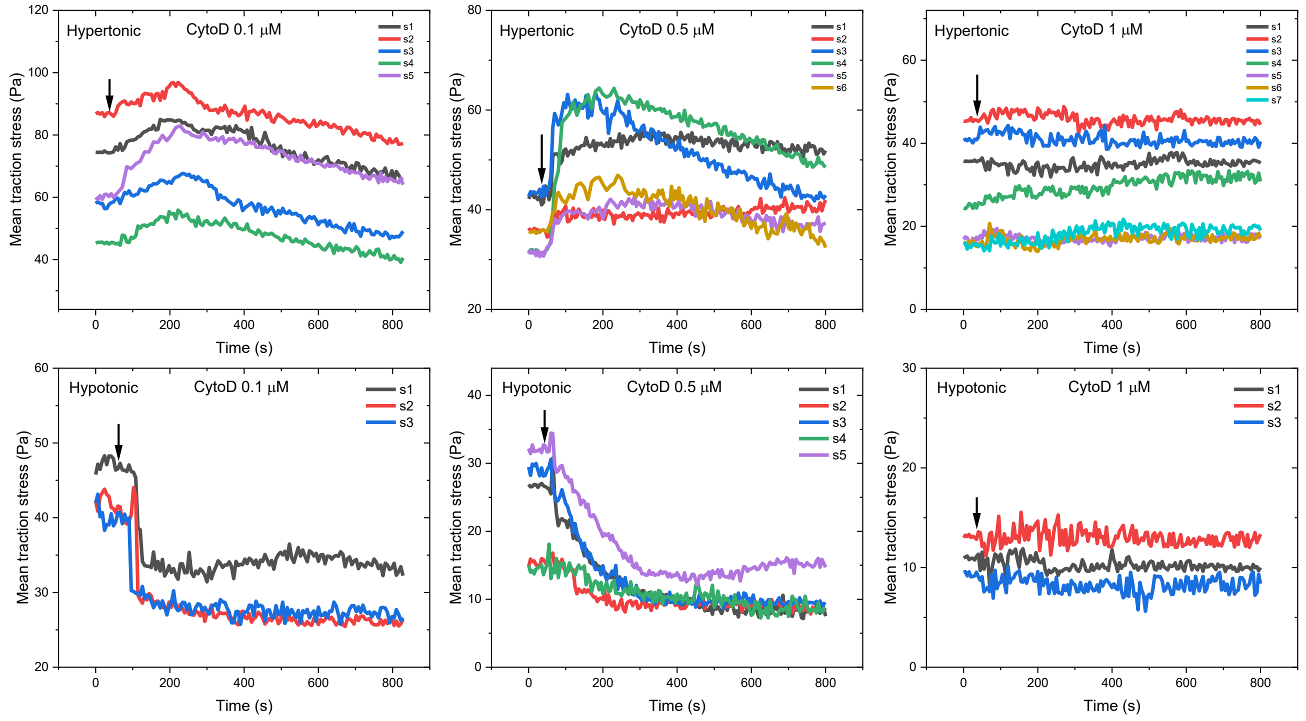

Figure S7: Time course of the traction stress (original data) exerted by cells treated with cytochalasin D (CytoD) under hypertonic and hypotonic shock. The black arrows indicate the onset of the osmotic shock.

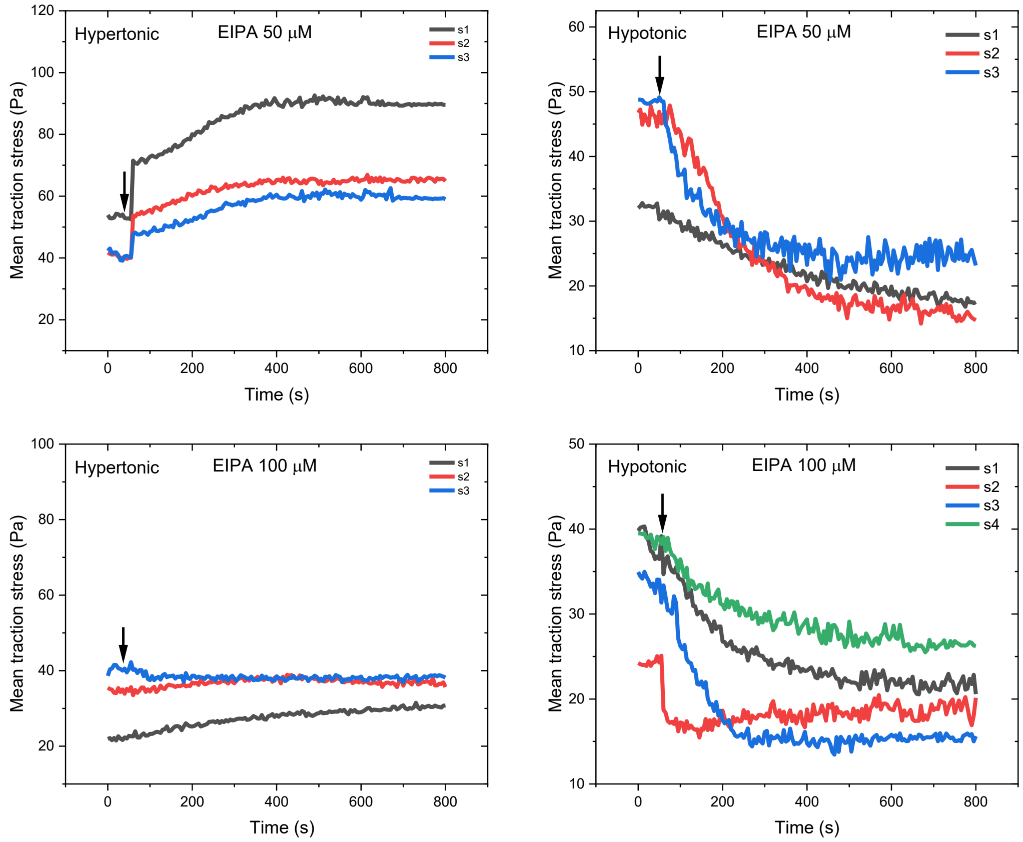

Figure S8: Time course of the traction stress (original data) exerted by cells treated with ethylisopropylamiloride (EIPA) under hypertonic and hypotonic shock. The black arrows indicate the onset of the osmotic shock.

3 Materials and Methods

3.1 Materials and reagents

3-Aminopropyltrimethoxysilane (APTES) and N-hydroxysuccinimide (NHS) were purchased from Sigma-Aldrich (St. Louis, MO, USA). Glass-bottom culture dishes were from Nest Biotechnology Co. Ltd. (Wuxi, China). Phosphate-buffered saline (PBS; 0.1 M phosphate buffer containing 0.9% sodium chloride, pH 7.5) and N-(3-*dimethylaminopropyl*)-N'-*ethylcarbodiimide* hydrochloride (EDC) were provided by Sangon Biotech Co. Ltd. (Shanghai, China). Carboxylate-modified green fluorescent beads (0.49 μm in diameter, 505/515 nm, F-8813, Molecular Probe) were obtained from Life Technologies (USA). Rat-tail tendon collagen type I was purchased from Shanghai Canspec Scientific Instruments Co. Ltd. (Shanghai, China). Sulfosuccinimidyl 6-(4′-azido-2′-nitrophenylamino) hexanoate, Dil and Hoechst 33258 were obtained from Thermo Fisher Scientific (sulfo-SANPAH, Waltham, MA, USA). Cytochalasin D (CytoD) was purchased from Shanghai Yuanye Bio-Technology Co., LTD (Shanghai, China). Ethylisopropylamiloride (EIPA) was purchased from Shanghai Macklin Biochemical Technology Co., Ltd (Shanghai, China). All other chemicals were from Beijing Chemical Reagents Co. Ltd. (Beijing, China). The circular quartz plate (12 mm diameter) was acquired from www.Taobao.com.

3.2 Osmotic shock

For osmotic experiments, the osmotic pressure of the medium was changed from isotonic (300 mOsm) to hypertonic (500 mOsm) and hypotonic (200 mOsm) during image acquisition, respectively. The hypertonic solution (500 mOsm) was obtained by adding mannitol to phosphate buffered saline (PBS). Mannitol was chosen because it is widely used as a high-osmotic antihypertensive agent clinically, such as in the treatment of intracranial hypertension [4] and intraocular pressure [5]. The hypotonic solution (200 mOsm) was obtained by adding water to PBS.

3.3 Drug treatments

After plating the cells on the substrate, cytochalasin D (CytoD, an inhibitor of actin polymerisation) and ethylisopropylamiloride (EIPA, an inhibitor of Na+/H+ antiporters) were added. Cells were incubated with the drugs for ∼50 min before imaging.

3.4 Cell culture

C2C12 cells and HepG2 cells (Shanghai Cell Bank of Chinese Academy of Sciences) were cultured in DMEM culture medium (Hyclone, Logan City, USA) supplemented with 10% fetal bovine serum (Gibco, Thermo Fisher Scientific Inc., UK), 100 U/mL penicillin G (Life Technologies, USA), and 100 μg/mL streptomycin (Life Technologies, USA). They were incubated at 37 °C. in a humidified 5% CO_2 atmosphere. Cells were detached from culture flasks with 0.25% trypsin solution (Gibco, USA) containing ethylenediaminetetraacetic acid (EDTA). The cells were centrifuged at 100 g for 5 min and resuspended in the culture medium. We then plated cells onto the gel substrate and incubated them for less than 1 h before imaging.

3.5 Substrate fabrication

The glass-bottom culture dish was pretreated with 4% (v/v) APTES solution before treated with 0.5% glutaraldehyde solution. Then the Petri dish was washed thoroughly with a stream of deionized water and dried in the air. The polyacrylamide (PAA) hydrogel was prepared using a protocol according to previous reports [6, 7]. Solutions of 4% (w/v) acrylamide, 0.15% (w/v) N,N-methylene-bis-acrylamide, 0.5% N, N, N', N'-tetramethylethylenediamine, and 0.05% ammonium persulfate were mixed to form the pre-gel solution. The pre-gel solution was spread onto the surface of a pre-cleaned circular quartz plate. Subsequently, the activated Petri dish was slowly inverted onto the top of the droplet from one side of the quartz plate. The acrylamide droplet between the Petri dish and the quartz plate became flat under gravity and polymerized for 60 min at room temperature. After removal of the Petri dish, the PAA gel was immersed in deionized water to remove the non-polymerized solution. The PAA gel, which was about 60-80 μm in thickness, was linked to the Petri dish along the bottom surface. The Young's modulus of the PAA gel was about 2.5 KPa [2, 8]. Next, 10 % (v/v) green fluorescent beads (2% (w/v), carboxylate-modified) together with deionized water was added to the modified PAA films for 40 min. To activate the carboxylic acid groups, a mixture of 0.5% NHS and 2% EDC in water was added to the Petri dish. The solution was left undisturbed in the dark light for 2.5 h. The beads were uniformly distributed on the surface of the substrate at a density of $\sim$1.5 beads/μm^2.

To promote cell adhesion on the gel surface, type I collagen was conjugated to the PAA film using the heterobifunctional cross-linker sulfo-SANPAH. 1 mg/mL solution of sulfo-SANPAH in water (200 μL) was pipetted onto the gel surface to activate PAA. The Petri dish was irradiated with UV light (10 W) for 10 min before it was covered with 0.6% (v/v) solution of collagen I in 6 mM aqueous acetic acid and incubated overnight at 4 °C.

3.6 Image acquisition

All images were collected with a Olympus 100×, NA 1.40 (numerical aperture) oil objective in an inverted Olympus confocal microscope (FV1000) equipped with a Olympus CCD camera. An argon laser (488 nm) was used to image the green fluorescent beads. To obtain traction force during osmotic shock, time-lapse images of cell and fluorescent beads were acquired every 3 seconds for 30 min at a resolution of 512 × 512 pixels, which corresponds to pixel dimensions of 0.12 × 0.12 μm. After the acquisition of the time-lapse images, SDS was used to remove cells from the substrate. And then the image of the beads were taken at the same position as un-deformed images. Cells were maintained in an incubation chamber (Tokai Hit, Shizuoka, Japan) at 37 °C with 5% CO_2. Cells stained were imaged at 550 nm for cell membrane and 360 nm for nuclei. Typical 3D images of cells were acquired at 512 × 512 × 100 voxels.

3.7 Cell volume measurement during osmotic shock

Cells were cultured on PAA gel and stained with Dil. Z-stacks (1 μm steps) were acquired by a Nikon Yokogawa CSU-W1 Spinning Disk Confocal Microscope (Nikon, Japan) equipped with a 100×, NA 1.45 (numerical aperture) oil objective (Nikon, Japan). Time-lapse images were captured every 20 seconds for 20 min. Osmotic solution (hypertonic or hypotonic solution) was added into culture dish during imaging. Cell volume and cell area were obtained from the confocal images in ImageJ.

3.8 Calculations for displacements, stress and force

All displacements were calculated based on images of beads using digital image correlation in the PMLAB software (PMLAB software; Nanjing PMLAB Sensor Tech Co., Ltd., Nanjing, China). The accuracy of digital image correlation is 0.05 pixel [9, 10]. The displacements in Figure 3 was normalized to a range of 0 to 1. All traction stress were reconstructed from the displacements and the constitution relation of the substrate on the basis of elastic mechanics theory in ABAQUS software, as previous works reported [1, 3].

Mean traction stress was obtained by algebraically averaging the total stress in the traction stress map,

$$\overline{F} = \frac{1}{S} \iint_{\Sigma} \sqrt{F_x(x,y)^2 + F_y(x,y)^2} \mathrm{d}x\mathrm{d}y \tag{1}$$

where $\overline{F}$ is the average traction stress, F_x and F_y are the x and y components of the traction stress, Σ is the region where a cell interacts with the substrate, S is the area of the Σ.

3.9 Calculations for cell cortical tension

The Ponder/Boyle/Vant'Hoff(PBVH) equation of state describes the relation between osmotic pressure and cell volume [11, 12]

$$P(V - V_{OI}) = P_0(V_0 - V_{OI}), \tag{2}$$

where P and V denote the osmotic pressure and cell volume, P_0 and V_0 are the osmotic pressure and cell volume under isotonic condition, and V_{OI} is the osmotically inactive volume. Roffay *et al.* describe the relation between osmotic pressure and cortical tension [11]

$$\frac{P}{P_0} = \frac{1 - \frac{V_{OI}}{V_0}}{\left[\frac{1+e^{(\mu-\sigma_0 a)/k_B T}}{e^{\mu/k_B T}+e^{\sigma a/k_B T}} e^{\sigma a/k_B T}\right]^{3/2} - \frac{V_{OI}}{V_0}}, \tag{3}$$

where μ is free energy, a is membrane area, σ is the cortical tension, σ_0 is the cortical tension under isotonic condition, k_B is the Boltzmann constant, and T is the absolute temperature. To obtain the relation between cell volume and cortical tension, we combine with Eq.(2) and Eq.(3) to yield Eq.(4)

$$\frac{V}{V_0} = \left(\frac{1 + e^{(\mu-\sigma_0 a)/k_B T}}{e^{\mu/k_B T} + e^{\sigma a/k_B T}} e^{\sigma a/k_B T}\right)^{3/2}. \tag{4}$$

According to the literature [11], $\mu = 1.5 k_B T$, $\sigma_0 = 1.2 \times 10^{-4} N/m$, $a = 53\ nm^2$. V/V_0 is derived from experimental measurement. Eq.(4) was used to calculate the cortical tension during osmotic shock.

4 Model and simulation

To valid the mechanism proposed for the change of the traction force, we simulated substrate deformation induced by a single cell under osmotic shock. The simulation was carried out in ABAQUS using the static implicit finite element method.

4.1 Model of cell and ECM

At the initial state, cell adheres on the substrate and state equilibrium. The mechanical force of the cell is balanced by cortical tension, cell membrane tension, actomyosin cytoskeleton and hydrostatic pressure [13]. In this work, we simply the cell model. Firstly, we treat cell membrane and cortex as a single layer to consider the tension they provide. Then, we assume that the contractile force generated by acto-mysoin on the contact surface is a constant. Cell cortex is subjected to hydrostatic pressure difference (the hydrostatic pressure inside the cell is larger than it in the extracellular environment) during osmotic shock[14, 15]. The hydrostatic pressure difference across cell membrane is balanced with the contractile force generated by mysoin on the actin filament network and the bond between cell and the substrate. Finally, we applied hydrostatic pressure difference on the cell cortex and regulated its value to simulate the osmotic pressure change.

Table S1: Summary of parameters used in simulation

Parameter	Values from experiment	Values in simulation
Thickness of cell cortex	0.05-1 μm [16]	1 μm
Elastic modulus of cell cortex	0.1-100 kPa [17]	3.5 kPa
Elastic modulus of the substrate	2.5 kPa [2, 8]	2.5 kPa
Poisson's ratio of cell cortex	-	0.3
Poisson's ratio of the substrate	-	0.45
Osmotic pressure outside the cell	0.5 MPa [18]	0.5 MPa
Contraction stress exerted by actin filaments	-	1 kPa

References

[1] Yongman Liu, Jianye Wang, Yong Su, Xiaohai Xu, Hong Liu, Kainan Mei, Shihai Lan, Shubo Zhang, Xiaoping Wu, Yunxia Cao, et al. Quantifying 3d cell-matrix interactions during mitosis and the effect of anticancer drugs on the interactions. *Nano Research*, 14:4163–4172, 2021.

[2] Aleksandra K Denisin and Beth L Pruitt. Tuning the range of polyacrylamide gel stiffness for mechanobiology applications. *ACS applied materials & interfaces*, 8(34):21893–21902, 2016.

[3] Martin Bergert, Tobias Lendenmann, Manuel Zündel, Alexander E Ehret, Daniele Panozzo, Patrizia Richner, David K Kim, Stephan JP Kress, David J Norris, Olga Sorkine-Hornung, et al. Confocal reference free traction force microscopy. *Nature communications*, 7:12814, 2016.

[4] Angelo Pascarella, Lucia Manzo, and Francesco Bono. Effect of mannitol bolus administration on cerebrospinal fluid pressure in patients with idiopathic intracranial hypertension: a pilot study. *Journal of Neurology*, 269(11):6158–6164, 2022.

[5] Varsha Ramachandra, Premanand Chandran, Reji Philip, Vinoth Arunaachalam, and Ganesh V Raman. Effect of mannitol on intraocular pressure in vitrectomized and nonvitrectomized eyes: a prospective comparative study. *Journal of Glaucoma*, 28(4):318–320, 2019.

[6] Robert J Pelham and Yu-li Wang. Cell locomotion and focal adhesions are regulated by substrate flexibility. *Proceedings of the National Academy of Sciences*, 94(25):13661–13665, 1997.

[7] Aleksandar Marinković, Justin D Mih, Jin-Ah Park, Fei Liu, and Daniel J Tschumperlin. Improved throughput traction microscopy reveals pivotal role for matrix stiffness in fibroblast contractility and tgf-β responsiveness. *American Journal of Physiology-Lung Cellular and Molecular Physiology*, 303(3):L169–L180, 2012.

[8] Justin R Tse and Adam J Engler. Preparation of hydrogel substrates with tunable mechanical properties. *Current protocols in cell biology*, 47(1):10–16, 2010.

[9] Xiaohai Xu, Yong Su, Yulong Cai, Teng Cheng, and Qingchuan Zhang. Effects of various shape functions and subset size in local deformation measurements using dic. *Experimental Mechanics*, 55(8):1575–1590, 2015.

[10] Xiaohai Xu, Yong Su, and Qingchuan Zhang. Theoretical estimation of systematic errors in local deformation measurements using digital image correlation. *Optics and Lasers in Engineering*, 88:265–279, 2017.

[11] Chloé Roffay, Guillaume Molinard, Kyoohyun Kim, Marta Urbanska, Virginia Andrade, Victoria Barbarasa, Paulina Nowak, Vincent Mercier, José García-Calvo, Stefan Matile, Robbie Loewith, Arnaud Echard, Jochen Guck, Martin Lenz, and Aurélien Roux. Passive coupling of membrane tension and cell volume during active response of cells to osmosis. 118(47), 2021.

[12] John D. Finan, Kevin J. Chalut, Adam Wax, and Farshid Guilak. Nonlinear Osmotic Properties of the Cell Nucleus. 37(3):477–491, 2009.

[13] Michele A Wozniak and Christopher S Chen. Mechanotransduction in development: a growing role for contractility. *Nature reviews Molecular cell biology*, 10(1):34–43, 2009.

[14] Martin P Stewart, Jonne Helenius, Yusuke Toyoda, Subramanian P Ramanathan, Daniel J Muller, and Anthony A Hyman. Hydrostatic pressure and the actomyosin cortex drive mitotic cell rounding. *Nature*, 469(7329):226–230, 2011.

[15] Hongyuan Jiang and Sean X Sun. Cellular pressure and volume regulation and implications for cell mechanics. *Biophysical journal*, 105(3):609–619, 2013.

[16] Guillaume Salbreux, Guillaume Charras, and Ewa Paluch. Actin cortex mechanics and cellular morphogenesis. *Trends in cell biology*, 22(10):536–545, 2012.

[17] Tatyana G Kuznetsova, Maria N Starodubtseva, Nicolai I Yegorenkov, Sergey A Chizhik, and Renat I Zhdanov. Atomic force microscopy probing of cell elasticity. *Micron*, 38(8):824–833, 2007.

[18] Jean-Yves Tinevez, Ulrike Schulze, Guillaume Salbreux, Julia Roensch, Jean-François Joanny, and Ewa Paluch. Role of cortical tension in bleb growth. *Proceedings of the National Academy of Sciences*, 106(44):18581–18586, 2009.
